# Relationship of breast volume, obesity and central obesity with different prognostic factors of breast cancer

**DOI:** 10.1038/s41598-021-81436-9

**Published:** 2021-01-21

**Authors:** Daniel María Lubián López, Carmen Aisha Butrón Hinojo, María Castillo Lara, Manuel Sánchez-Prieto, Rafael Sánchez-Borrego, Nicolas Mendoza Ladrón de Guevara, Ernesto González Mesa

**Affiliations:** 1 Department of Obstetrics and Gynecology, University Hospital Jerez de La Frontera, Hospital Quirónsalud Campo de Gibraltar, Faculty of Medicine, University of Cádiz, Cádiz, Spain; 2 Department of Obstetrics and Gynecology, Hospital Punta de Europa, Algeciras, Cádiz, Spain; 3 Department of Obstetrics and Gynecology, University Hospital Puerto Real, Cádiz, Spain; 4 Department of Obstetrics and Gynecology, Institut Universitari Dexeus, Barcelona, Spain; 5 Department of Obstetrics and Gynecology, DIATROS, Women’s Care Clinic, Barcelona, Spain; 6 Department of Obstetrics and Gynecology, University of Granada, Granada, Spain; 7 Department of Surgical Specialties, Biochemistry and Immunology, University Hospital of Malaga, Faculty of Medicine, University of Malaga, Málaga, Spain

**Keywords:** Surgical oncology, Endocrinology, Medical research, Oncology, Risk factors

## Abstract

The objective of this study was to investigate whether the BC tumor biology in women with larger breast volume, in obese women and especially in women with central adiposity at the moment of diagnosis of BC is more aggressive than in those women without these characteristics. 347 pre- and postmenopausal women with a recent diagnosis of BC were analyzed. In all patients, anthropometric measurements at the time of diagnosis was collected. In 103 of them, the breast volume was measured by the Archimedes method. The Breast volume, BMI, WHR and the menopausal status were related to different well-known pathological prognostic factors for BC. At the time of diagnosis, 35.4% were obese (BMI > 30 kg/m^2^), 60.2% had a WHR ≥ 0.85, 68.8% were postmenopausal and 44.7% had a breast volume considered "large" (> 600 cc). Between patients with a large breast volume, only a higher prevalence of ER (+) tumors was found (95.3% vs. 77.2%; *p* = 0.04) compared to those with small breast volumes. The obese BC patients showed significantly higher rates of large tumors (45.5% vs. 40.6%; *p* = 0.04), axillary invasion (53.6% vs. 38.8%; *p* = 0.04), undifferentiated tumors (38.2% vs. 23.2%) and unfavorable NPI (*p* = 0.04) than non-obese women. Those with WHR ≥ 0.85 presented higher postsurgical tumor stages (61.7% vs. 57.8%; *p* = 0.03), higher axillary invasion (39.9% vs. 36.0%; *p* = 0.004), more undifferentiated tumors (30.0% vs. 22.3%; *p* = 0.009), higher lymphovascular infiltration (6.5% vs. 1.6%; *p* = 0.02), and a higher NPI (3.6 ± 1.8 vs. 3.2 ± 1.8; *p* = 0.04). No statistically significant differences were found according to menopausal status. We conclude that obesity, but especially central obesity can be associated with a more aggressive tumour phenotype. No relation between breast volume and tumoral prognostic factors was found, except for a higher proportion of ER (+) tumor in women with higher breast volume.

## Introduction

There is a growing interest in the association between obesity and breast cancer (BC). Epidemiological data have revealed an association of increased BC incidence and mortality with obesity, particularly in postmenopausal women^1^. Some recognized anthropometric factors influence BC prognosis. A high body mass index (BMI) is associated with worse prognosis in premenopausal and postmenopausal women^2–7^.

Some studies have revealed a worse BC prognosis (greater axillary involvement and shorter disease-free survival) among obese postmenopausal women than among nonobese women^5,8^. It is still unclear whether a higher BMI is associated with positive estrogen receptor (ER) or progesterone receptor (PR) BC. Some, but not all^9^, studies^10,11^ have suggested a higher percentage of ER (+) BC among obese postmenopausal patients than among nonobese patients.

Abdominal (waist) circumference is a parameter used to measure and diagnose central obesity, which is closely related to the prognosis of BC. Consequently, a high waist circumference is associated with a worse BC prognosis in premenopausal and postmenopausal women^12,13^. In addition, a high waist circumference has been associated with an advanced histological grade in postmenopausal patients and with larger tumors in premenopausal women^12,13^.

After adjusting for BMI, an association between a higher waist–hip ratio (WHR) and worse prognosis of BC has been suggested in patients with premenopausal BC but not in those with postmenopausal BC^14^. However, other authors have described a higher WHR as an independent poor prognostic factor in ER-positive postmenopausal women after adjusting for BMI^15^. A high WHR can be used as an indirect marker of a high testosterone/estrogen ratio and, possibly and most importantly, of insulin resistance and high fasting insulin, pro-insulin and C-peptide levels in women^16–18^. Hyperinsulinemia could also be associated with a worse outcome in advanced BC patients^19–21^.

In some studies, the size and volume of the breast have been associated with more aggressive characteristics of the tumor at the time of diagnosis in pre- and postmenopausal women^22–26^. Although breast size is strongly correlated with BMI^27^, only one-third of the genes that contribute to breast size have been shown to influence BMI^28^. In young women who are nonusers of oral contraceptives, during the follicular phase, breast size was significantly positively associated with insulin-like growth factor-1^29^. In addition, a large breast size at age 20 has been described as a predictor of type 2 diabetes mellitus in middle-aged women, even after adjusting for BMI and WHR^30^. Patients with ER-negative BC and type 2 diabetes have a higher risk of metastasis and mortality than patients without diabetes, but this was not observed in patients with ER-positive BC^31^.

Studies that investigate the size of the breast in relation to BC have frequently used bra cup size (A, B and C) as a reference measure^24,28,32^. However, different manufacturers have an inconsistent bra cup sizes^33^. On the other hand, the use of cup size alone without taking rib cage circumference into account is a poor surrogate for actual breast volume, even when BMI is taken into consideration^33^. The measurement of breast volume using plastic cubes used by plastic surgeons performing breast reductions and reconstructions leads to more reproducible results^34^. This system, based on Archimedes' method, has been rarely used and allows us to better evaluate whether the breast volume itself has an impact on the type or growth pattern of the tumor. The identification of prognostic factors makes it possible to better adapt the treatment and can also help to identify pathophysiological pathways and therapeutic innovations in this area.

The goal of this study was to investigate whether the BC tumor biology in women with larger breast volume, in obese women and especially in women with central adiposity at the moment of diagnosis of BC is more aggressive than in those women without these characteristics.

## Materials and methods

A cross-sectional study was carried out in Caucasian pre- and postmenopausal women with BC. Before surgical treatment for their primary BC, in all women, a gynecological and nutritional history was performed, and anthropometric measurements and breast volume measurements were performed. The patients were excluded if they had a previous history of breast plastic surgery (mammoplasties to increase volume or reduction surgery) and/or history of breast‐conserving surgery (that deformed the breast), had previously undergone abdominoplasty surgery, were receiving neoadjuvant therapy currently or received it in the last 12 months, were receiving or had received any hormonal therapy (HT) during the last 12 months, had gone on a strictly restricted diet in the last 12 months, had lost > 3 kg in the last year, suffered from carcinoma in situ (ductal or lobular carcinoma), claimed to not understand the object of the investigation, or did not sign the informed consent form in order to take part in the study.

Weight was determined with a tested precision electronic scale that displayed weight in 0.1 kg (kg) increments; the patients did not wear heavy clothes or shoes. Height was determined in 0.5-cm (cm) increments with the patient barefoot on a stadiometer. Body mass index (BMI) = mass (kg)/height (m^2^), and obesity was defined as a Quetelet Index ≥ 30 kg/m^2^ according to the World Health Organization (WHO) definition. Waist circumference was measured using a plastic tape measure with metric graduation and a minimum increment of 1 mm (mm). This tape measure was placed at the midpoint between the lowest rib and the iliac crest, with the patient standing after gentle expiration. Hip circumference was measured by placing the tape measure around the top of the hips and buttocks at the widest point. Waist-hip ratio (WHR) was calculated in all women. A WHR ≥ 0.85 indicated central obesity. The breast volume was measured by the Archimedes method by introducing the breast in a container with warm water and measuring the volume of the displaced water. In fluid mechanics, we speak of displacement (or dislodged volume) when an object is immersed in a fluid and displaces it. The volume of the displaced fluid can be measured, and from this, the volume of the submerged body can be deduced (which must be exactly equal to the volume of the dislodged fluid). Displacement can be used to measure the volume of a solid object, even if its shape is not regular. We have used the method by which the object (the breast) is immersed in a completely filled container of water, causing it to spill over. Then, the spilled water is collected in another larger container placed below the previous container, and its volume is measured, which will be equal to the volume of the object introduced (the breast). All measurements were taken by the same observer to reduce intraobserver error. Large breasts are considered when they have a volume > 600 cc (median). Women with amenorrhea ≥ 1 year and FSH levels > 40 UI/l were defined as menopausal.

BC was classified pathologically using a modified version of the Elston Ellis of the Scarff Bloom Richardson grading system. Clinical classification was carried out according to the Classification of Malignant Tumors (TNM). The study of estrogen receptors (ERs), progesterone receptors (PRs), c-erbB2 and Ki-67 was carried out through immunohistochemistry techniques. Triple-negative tumors (TNs) were defined as ER negative, PR negative, and c-erbB2 negative. In the BC patients who needed neoadjuvant treatment (chemotherapy and/or endocrine treatment), all the pathological factors of the tumor were determined from the previous diagnostic biopsy, except the possible axillary affectation, which was evaluated as negative in the clinical exploration or positive if fine needle aspiration biopsy or core needle biopsy of axillary adenopathy was performed before neoadjuvant treatment.

This study was conducted according to the guidelines of the Declaration of Helsinki and resolution 196/96 of the National Health Council on Research Involving Human Subjects^35^. Approval was obtained from our hospital ethics committee (Research ethics committee of Cadiz/CEI/15032016).

### Statistical analysis

Data were compiled and analyzed using SPSS 15.0 for Windows (11.5 version, SPSS Inc., USA). All data are expressed as the mean ± standard deviation. Clinical and anthropometric variables of patients were compared between the two different groups of women (small vs. large breast; obese vs. nonobese, central obesity vs. central nonobesity, pre- vs. postmenopausal status). Prognostic tumor characteristics in patients with BC were analyzed according to their breast volume, BMI, WHR, and menopausal status. The relationship between breast volume and BMI in all patients with breast cancer was calculated. Statistical analysis was carried out by calculating frequencies, means and standard deviations. Generally, percentages are reported in relation to responses to specific questions and may vary between items. Chi-square or Fisher's exact tests were adopted for comparisons of frequencies, and Student's t-test was used for comparisons of means. The nonparametric Mann–Whitney U-test or Kruskal–Wallis test (when appropriate) was used to assess the differences in the distribution of the prognostic factors in the different groups. The bivariate correlation coefficient of Pearson's r was used to determine whether there was a linear relationship between breast volume and age, BMI or WHR. Statistical significance was indicated by a *p* value < 0.05.

### Research involving human participants and/or animals

This study was conducted according to the guidelines of the Helsinki Declaration and resolution 196/96 of the National Health Council on Research Involving Human Subjects. Approval of our hospital ethics committee was obtained (Research ethics committee of Cadiz/CEI/15032016).

### Informed consent

All participants signed an informed consent before taking part in the study.

## Results

The study included 402 consecutively enrolled patients; 365 (90.7%) did not meet the exclusion criteria, and 347 agreed to take part (participation rate of 86.3%). For the study of breast volume, information was only obtained for 103 patients (103/347 = 29.68%).

Of these women, at the time of diagnosis, 35.4% were obese (BMI > 30 kg/m^2^), 64.5% were nonobese, 60.2% had a WHR ≥ 0.85, 39.8% had a WHR < 0.85, 68.8% were postmenopausal, and 31.1% were premenopausal. Of the 103 patients assessed for this variable, 44.7% had a breast volume considered "large" (> 600 cc), compared to 55.3% with "small volume” breasts (< 600 cc). There were no systematic differences in age, TNM classification, or the use of adjuvant endocrine treatment between BC patients who participated and those who declined participation (data not shown).

The mean age of the patients was 59.09 ± 12.85 years. In our setting, obese women with a high WHR and postmenopausal women with breast cancer were older and had more children than nonobese women with a lower WHR and premenopausal status (Table 1).

**Table 1:** Demographic characteristics of patients with breast cancer according to their breast volume (small vs. large), BMI (obesity vs. non-obesity), central obesity (WHR < 0.85 vs. WHR ≥ 0.85) and hormonal status (premenopausal vs. postmenopausal).

Clinical variable	Breast volume				Obesity			
Demographic variable	All patients n = 103 (100%)	Small n = 57 (55.33%)	Large n = 46 (44.66%)	*p*	All patients n = 347 (100%)	No n = 224 (64.55%)	Yes n = 123 (35.45%)	*p*
Age (years)	58.05 ± 12.70	57.36 ± 13.01	58.65 ± 12.34	0.60	59.09 ± 12.85	55.70 ± 12.42	65.28 ± 11.27	0.00*
Age of menarche (years)	12.69 ± 1.53	12.68 ± 1.22	12.73 ± 1.84	0.87	12.69 ± 1.65	12.56 ± 1.47	12.85 ± 1.78	0.12
Age of menopause (years)	49.11 ± 5.02	49.39 ± 4.23	49.00 ± 6.03	0.76	49.33 ± 4.85	49.15 ± 4.40	49.57 ± 5.39	0.52
Years since menopause	16.34 ± 10.93	15.94 ± 6.75	16.97 ± 3.72	0.86	16.34 ± 10.93	16.01 ± 6.32	16.68 ± 3.70	0.55
Gestations	3.03 ± 2.74	3.05 ± 2.64	3.65 ± 2.85	0.99	2.95 ± 2.3	2.48 ± 1.67	3.82 ± 3.01	0.00*

Clinical variable	Central obesity				Menopausal status			
Demographic variable	All patients n = 304 (100%)	No n = 121 (39.80%)	Yes n = 183 (60.20%)	*p*	All patients n = 347 (100%)	Premenopausal n = 108 (31.12%)	Postmenopausal n = 239 (68.88%)	*p*
Age (years)	59.02 ± 11.65	54.80 ± 12.45	61.74 ± 12.43	0.00*	59.09 ± 12.85	44.61 ± 4.75	65.64 ± 9.59	0.00*
Age of menarche (years)	12.72 ± 1.55	12.71 ± 1.490.8	12.74 ± 1.58	0.84	12.69 ± 1.65	12.43 ± 1.39	12.77 ± 1.66	0.10
Age of menopause (years)	49.18 ± 5.01	49.10 ± 5.20	49.22 ± 4.80	0.86	49.33 ± 4.85	–	49.33 ± 4.85	–
Years since menopause	16.17 ± 9.73	16.97 ± 3.72	15.78 ± 2.73	0.78	16.34 ± 10.93	–	16.34 ± 10.93	–
Gestations	2.96 ± 2.2	2.52 ± 1.71	3.34 ± 2.72	0.00*	2.95 ± 2.3	2.12 ± 1.13	3.32 ± 2.59	0.00*

n = number of cases assessed for each variable. Data expressed as means ± standard deviation.

*p < 0.05.

### Anthropometry according to breast volume, BMI, WHR and menopausal status

The anthropometric differences between the patients according to their breast volumes, BMI, WHR and menopausal status are shown in Table 2.

**Table 2:** Anthropometric characteristics of the patients with breast cancer according to their breast volume (small vs. large), BMI (obesity vs. non-obesity), central obesity (WHR < 0.85 vs. WHR ≥ 0.85) and hormonal status (premenopausal vs. postmenopausal).

Clinical variable	Breast volume				Obesity			
Anthropometric variable	All patients n = 103 (100%)	Small n = 57 (55.33%)	Large n = 46 (44.66%)	*p*	All patients n = 347 (100%)	No n = 224 (64.55%)	Yes n = 123 (35.45%)	*p*
Weight (kg)	72.56 ± 15.21	63.92 ± 10.54	81.35 ± 18.34	0.00*	71.76 ± 9.21	63.46 ± 7.67	85.50 ± 12.16	0.00*
Size (cm)	158.34 ± 4.54	157.64 ± 4.99	160.48 ± 7.17	0.30	158.84 ± 6.54	159.18 ± 6.48	156.17 ± 7.29	0.00*
Waist (cm)	94.54 ± 13.65	88.22 ± 12.07	101.68 ± 15.06	0.00*	92.76 ± 11.45	87.17 ± 12.71	107.26 ± 10.89	0.00*
Hip (cm)	107.56 ± 11.21	102.04 ± 9.21	113.89 ± 13.26	0.00*	105.87 ± 9.45	101.98 ± 7.75	118.50 ± 9.10	0.00*
Body mass index (kg/m^2^)	28.98 ± 5.76	25.57 ± 4.67	31.43 ± 6.92	0.00*	27.99 ± 2.99	24.91 ± 2.88	34.88 ± 3.84	0.00*
Waist/hip ratio (WHR)	0.87 ± 0.74	0.86 ± 0.71	0.89 ± 0.79	0.03	0.86 ± 0.44	0.85 ± 0.10	0.90 ± 0.80	0.00*

Clinical variable	Central obesity				Menopausal status			
Anthropometric variable	All patients n = 304 (100%)	No n = 121 (39.80%)	Yes n = 183 (60.20%)	*p*	All patients n = 347 (100%)	Premenopausal n = 108 (31.12%)	Postmenopausal n = 239 (68.88%)	*p*
Weight (kg)	71.44 ± 7.20	66.61 ± 12.61	75.22 ± 14.37	0.00*	71.74 ± 9.20	66.89 ± 13. 17	73.18 ± 14.22	0.00*
Size (cm)	158.06 ± 2.50	159.85 ± 7.00	157.38 ± 6.76	0.02	159.01 ± 6.50	161.79 ± 6.40	156.44 ± 6.44	0.00*
Waist (cm)	93.06 ± 13.43	83.65 ± 10.39	101.79 ± 14.00	0.00*	92.78 ± 11.43	86.79 ± 12.53	98.14 ± 15.46	0.00*
Hip (cm)	107.34 ± 7.55	104.83 ± 10.91	110.20 ± 11.38	0.00*	105.89 ± 9.65	102.81 ± 10.68	110 ± 11.07	0.00*
Body mass index (kg/m^2^)	28.38 ± 3.22	25. 99 ± 4.72	30.23 ± 5.70	0.00*	28.01 ± 3.02	25.38 ± 4.53	29.80 ± 7.73	0.00*
Waist/hip ratio (WHR)	0.87 ± 0.45	0.79 ± 0.04	0.92 ± 0.09	0.00*	0.86 ± 0.47	0.84 ± 0.07	0.88 ± 0.10	0.00*

n = number of cases assessed for each variable. Data expressed as means ± standard deviation.

*p < 0.05.

The average BMI was 28.98 ± 5.76, which was significantly greater in the group with a higher breast volume (31.43 ± 6.92 kg/m^2^ vs. 25.57 ± 4.67 kg/m^2^ in small breast; *p* = 0.000). The mean WHR was 0.87 ± 0.74, which was also significantly greater in the group with large breasts (0.89 ± 0.79 vs. 0.86 ± 0.71 in the group with small volume breasts; *p* = 0.03) (Table 2).

As expected, weight, waist circumference, hip circumference, BMI and WHR were higher in obese patients and in patients with a high WHR. Postmenopausal patients also had a significantly higher BMI (29.80 ± 7.73 vs. 25.38 ± 4.53 in premenopausal women; *p* = 0.000), and the WHR was also significantly higher than that of premenopausal women (0.88 ± 0.10 vs. 0.84 ± 0.07) (Table 2).

Breast volume was significantly higher in obese patients than in nonobese patients (868.12 ± 338.65 vs. 471.38 ± 239.07; *p* = 0.000) and in patients with a high WHR (652.84 ± 344.18 vs. 473.14 ± 242.75; *p* = 0.000). Although the postmenopausal women also presented more voluminous breasts (631.23 ± 338.14 vs. 511.61 ± 305.32; *p* = 0.08), the differences were not significant (Table 3).

**Table 3:** Breast volume in breast cancer patients according to their BMI (obese vs. non-obese), to their WHR (< 0.85 vs ≥ 0.85) and to their hormonal status (pre- vs. postmenopausal).

Clinical variable	Obesity				Central obesity				Hormonal status			
	All patients N = 103 (100%)	No n = 71 (68.93%)	Yes n = 32 (31.06%)	*p*	All patients n = 103 (100%)	No n = 36 (34.95%)	Yes n = 67 (65.04%)	*p*	All patients n = 103 (100%)	Premenopausal n = 35 (34.98%)	Postmenopausal n = 68 (66.01%)	*p*
Breast volume (Archimedes) (cc)		471.38 ± 239.07	868.12 ± 338.65	0.00*		473.14 ± 242.75	652.84 ± 344.18	0.00*		511.61 ± 305.32	631.23 ± 338.14	0.08

n = number of cases assessed for each variable. Data expressed as means ± standard deviation.

*p < 0.005.

**Figure 1:**
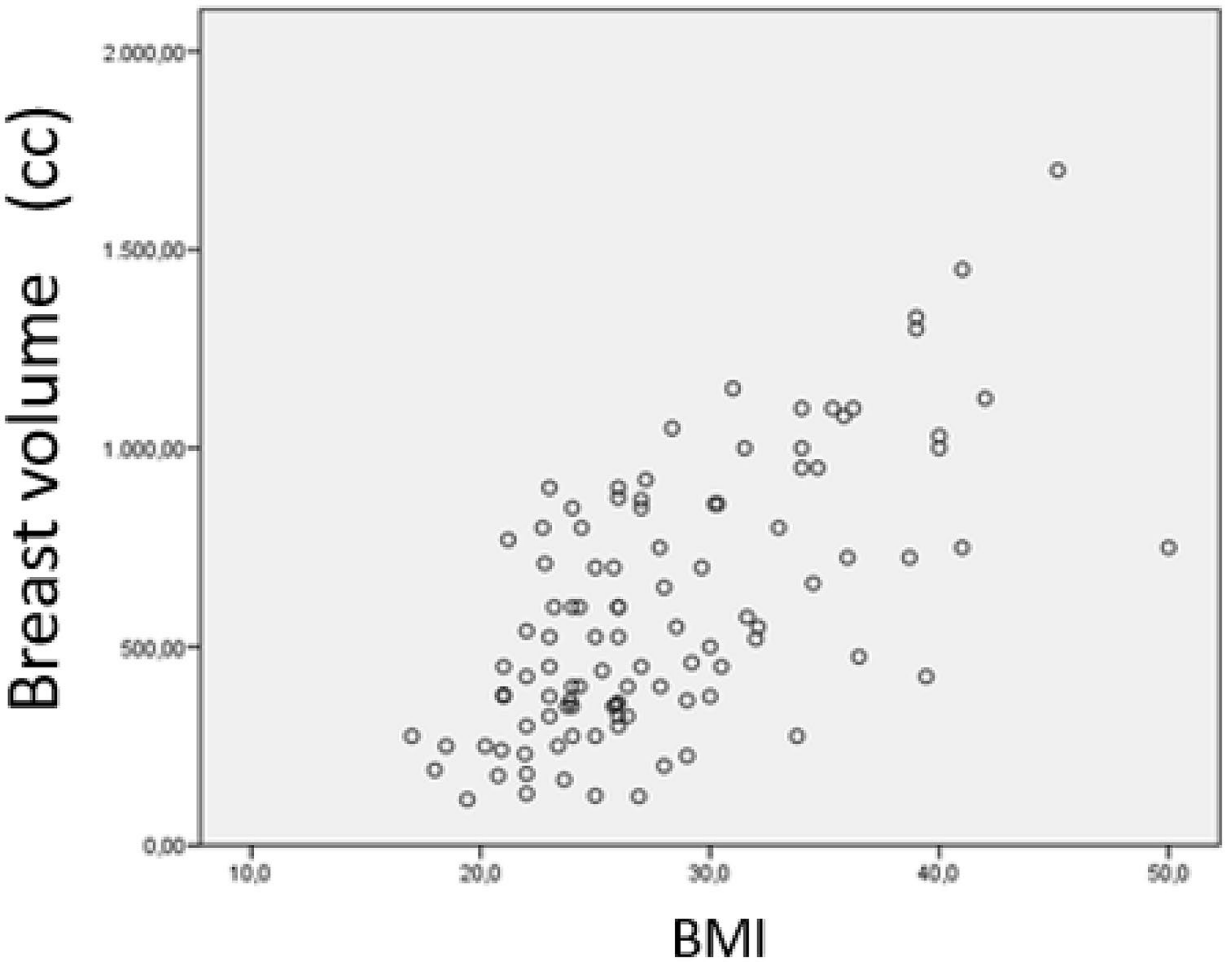
Relationship between Breast Volume and BMI in all patients with breast cancer. Pearson correlation coefficient. r = 0.65; (*p* = 0.000). Breast volume (cc): breast volume measured by Archimedes' method (cubic centimeters). BMI: body mass index.

There was a statistically significant correlation between breast volume and patient age (r = 0.20; *p* = 0.04) and WHR (r = 0.24; *p* = 0.01) (data not shown), and the correlation was even stronger with BMI (r = 0.65; *p* = 0.000) (Fig. 1).

### Relationships of breast volume, BMI, WHR and menopausal status with prognostic factors for breast cancer

Among the patients with a large breast volume, only a higher prevalence of ER (+) tumors was found (91.3% vs. 77.1%, *p* = 0.04) compared to those with small breast volumes (Table 4).

**Table 4:** Prognostic characteristics of tumor in breast cancer patients according to their breast volume (small vs. large).

Variable	All patients n = 103 (100%)	Small volume n = 57 (55.3%)	Large volume n = 46 (44.6%)	*p*
Clinical tumor size (mm)	23.95 ± 16.46	23.07 ± 17.06	24.80 ± 16.02	0.65
Tumor size pathological measurement (mm)	23.80 ± 16.16	24.67 ± 18.53	22.70 ± 12.73	0.42
**Tumor size (examination) (cm)**				
≤ 2 cm	58 (56.31%)	35 (61.40%)	23 (50.00%)	0.19
> 2 cm	45 (43.68%)	22 (38.60%)	23 (50.00%)	
**Clinical stage**				
I	30 (37.97%)	18 (42.85% )	12 (32.43%)	0.66
II, III	41 (51.90%)	20 (47.61%)	21 (56.75% )	
III	8 (10.13%)	4 (9.52%)	4 (10.81%)	
**Pathological stage**				
I	36 (42.35% )	21 (44.68% )	15 (39.47%)	0.96
II, III	40 (47.06% )	21 (44.68% )	19 (50%)	
IV	9 (10.59% )	5 (10.64% )	4 (10.53%)	
**Axillary involvement**				
Negative	48 (58.54%)	26 (57.78%)	22 (59.46%)	0.87
Positive	34 (41.46%)	19 (42.22%)	15 (40.54%)	
**Histological type**				
Ductal	92 (96.48%)	51 (94.44%)	41 (100%)	0.17
Lobulillar	3 (3.16%)	3 (5.56%)	0	
**Differentiation grade**				
1, 2	53 (68.83% )	32 (74.42%)	21 (61.76% )	0.48
3	24 (31.17% )	11 (25.58% )	13 (38.24% )	
**Lymphovascular invasion (n = 77)**^**a**^				
Yes	74 (96.10%)	41 (97.62%)	33 (94.29% )	0.72
No	3 (3.9%)	1 (2.38%)	2 (5.71% )	
**Ki-67 (n = 84)**^**a**^				
(+)	75 (89.29%)	39 (86.67%)	36 (92.31% )	0.52
(−)	9 (10.71%)	6 (13.33%)	3 (7.69% )	
Ki-67 (%) (n = 84)^a^	27.76 ± 22.3	27.96 ± 24.1	27.17 ± 20.3	0.93
**ER**				
(+)	76 (83.52%)	37 (77.00%)	39 (95.1%)	0.04*
(−)	15 (16.48%)	11 (22.92%)	2 (9.3%)	
**PR**				
(+)	71 (78.02%)	36 (75%)	35 (81.4%)	0.26
(−)	20 (21.98%)	12 (25%)	8 (18.6%)	
**Cerb2 (**^**a**^**)**				
(+)	24 (28.24%)	11 (24.44%)	13 (32.5% )	0.40
(−)	61 (71.76%)	34 (75.56%)	27 (67.5%)	
NPI	3.43 ± 1.78	3.34 ± 1.74	3.54 ± 1.84	0.62
**NPI**				
< 2	14 (17.07%)	8 (17.78%)	6 (16.22% )	0.13
2–2.4	16 (19.52%)	9 (20%)	7 (18.92% )	
2.4–3.4	14 (17.07%)	6 (13.33%)	8 (21.62% )	
3.4–5.4	17 (20.73%)	14 (31.11%)	3 (8.11% )	
> 5.4	21 (25.61%)	8 (17.78%)	13 (35.14% )	

Data expressed as means ± standard deviation and absolute numbers and their frequencies.

*NPI* Nottingham prognostic index.

*p value < 0.05 (t-Student or Chi-squared).

^a^Analysis performed on the cases provided by the pathology department.

In the obese patient group, we observed a higher proportion of tumors larger than 2 cm (45.5% vs. 40.6%; *p* = 0.04), a higher percentage of axillary involvement (53.6 vs. 38.8; *p* = 0.04), a higher proportion of undifferentiated tumors (38.2% vs. 23.2%) and higher rates of unfavorable NPIs (*p* = 0.04) than in the nonobese group (Table 5). Those with WHR ≥ 0.85 presented higher postsurgical tumor stages (61.7% vs. 57.8%; *p* = 0.03), higher axillary invasion (39.9% vs. 36.0%; *p* = 0.004), more undifferentiated tumors (30.0% vs. 22.3%; *p* = 0.009), higher lymphovascular infiltration (6.5% vs. 1.6%; *p* = 0.02), and a higher NPI (3.6 ± 1.8 vs. 3.2 ± 1.8; *p* = 0.04) (Table 6). No significant differences were found in any of the variables studied between pre- and postmenopausal women (Table 7).

**Table 5:** Prognostic tumor characteristics in breast cancer patients according to their BMI (obese vs. non-obese).

Variable	All patients n = 347 (100%)	Non-obese n = 224 (64.55%)	Obese n = 123 (35.45%)	*p*
Clinical tumor size (mm)	22.63 ± 14.64	21.48 ± 14.32	23.65 ± 15.13	0.36
Pathological tumor size (mm)	24.23 ± 14.87	23.93 ± 16.13	24.50 ± 14.56	0.76
**Tumor size (cm)**				
≤ 2 cm	151 (57.85%)	94 (59.49%)	57 (55.34%)	0.04*
> 2 cm	110 (42.15%)	64 (40.51%)	46 (44.66%)	
**Clinical stage**				
I	115 (41.22%)	75 (42.37%)	40 (39.22%)	0.66
II, III	143 (51.25%)	87 (49.16%)	56 (54.9%)	
IV	21 (7.53%)	15 (8.47%)	6 (5.88%)	
**Pathological stage**				
Initial (I)	111 (36.75%)	76 (40%)	35 (31.25%)	0.08
Intermediate (II, III)	168 (55.63%)	97 (51.05%)	71 (63.39%)	
Advanced (IV)	23 (7.62%)	17 (8.95% )	6 (5.36% )	
**Axillary involvement**				
Negative	172 (58.50%)	112 (61.20%)	54 (46.15%)	0.04*
Positive	122 (41.5%)	71 (38.80%)	63 (53.84%)	
**Histological type**				
Ductal	224 (94.12%)	138 (93.88%)	86 (94.5%)	0.69
Lobulillar	14 (5.88%)	9 (6.12%)	5 (5.49%)	
**Differentiation degree**				
1, 2	196 (71.01%)	131 (76.61%)	65 (61.9%)	0.01*
3	80 (28.99%)	40 (23.39%)	40 (38.1%)	
**Lymphovascular invasion (**^**a**^**)**				
Yes	14 (5.98%)	14 (9.4%)	0	0.13
No	220 (94.02%)	135 (90.6%)	85 (100%)	
**Ki-67 (**^**a**^**) (n = 84)**				
(+)	236 (86.45%)	151 (85.8%)	85 (87.63%)	0.28
(−)	37 (13.55%)	25 (14.2%)	12 (12.37%)	
Ki-67 (%) (^a^)	24.45 ± 5.10	27.69 ± 3.10	21.57 ± 7.54	0.23
**ER**				
(+)	269 (85.4% )	177 (86.76%)	92 (82.88%)	0.54
(−)	46 (14.6% )	27 (13.24%)	19 (17.12%)	
**PR**				
(+)	244 (78.46%)	159 (79.10%)	85 (77.27%)	0.85
(−)	67 (21.54%)	42 (20.9%)	25 (22.73%)	
**Cerb2 (**^**a**^**)**				
(+)	83 (29.12%)	57 (31.32%)	26 (25.24%)	0.44
(−)	202 (70.88%)	125 (68.68%)	77 (74.76%)	
NPI	3.63 ± 1.89	3.53 ± 1.82	3.88 ± 1.95	0.17
**NPI**				
< 2	49 (16.23%)	30 (16.30%)	19 (17.59%)	0.04*
2–2.4	55 (18.21%)	29 (15.76%)	16 (14.81%)	
2.4–3.4	58 (19.21%)	46 (25%)	12 (11.11%)	
3.4–5.4	53 (17.55%)	30 (16.30%)	23 (21.3%)	
> 5.4	87 (28.81%)	49 (26.63%)	38 (35.18%)	

Data expressed as means ± standard deviation and absolute numbers and their frequencies.

*NPI* Nottingham prognostic index.

*p value < 0.05 (t-Student or Chi-squared).

^a^Analysis performed on the cases provided by the pathology department.

**Table 6:** Prognostic tumor characteristics in patients with breast cancer according to WHR (central obesity vs. no central obesity).

Variable	All patients n = 304 (100%)	WHR < 0.85 n = 121 (%)	WHR ≥ 0.85 n = 183 (60.19%)	*p*
Clinical tumor size (mm)	23.22 ± 13.86	24.41 ± 18.72	21.64 ± 12.01	0.06
Pathological tumor size (mm)	22.95 ± 12.13	21.76 ± 15.24	23.48 ± 15.27	0.39
**Tumor size (cm)**				
≤ 2 cm	137 (60.62%)	54 (62.07%)	83 (59.71%)	0.11
> 2 cm	89 (39.38%)	33 (37.93%)	56 (40.29%)	
**Clinical stage**				
I	106 (43.80%)	44 (44.44%)	62 (43.36%)	0.24
II, III	118 (48.76%)	46 (46.46%)	72 (50.35%)	
III	18 (7.44%)	9 (9.09%)	9 (6.29%)	
**Pathological stage**				
Initial (I)	103 (39.77%)	42 (42.42%)	61 (38.13%)	0.03
Intermediate (II, III)	136 (52.51%)	47 (47.47%)	89 (55.63%)	
Advanced (IV)	20 (7.72%)	10 (10.11%)	10 (6.25%)	
**Axillary involvement**				
Negative	154 (60.87%)	58 (62.37%)	96 (60%)	0.00*
Positive	99 (39.13%)	35 (37.63%)	64 (40%)	
**Histological type**				
Ductal	263 (95.29%)	109 (95.61%)	154 (95.06%)	0.64
Lobulillar	13 (4.71%)	5 (4.39%)	8 (4.94%)	
**Differentiation degree**				
1, 2	172 (73.19%)	68 (77.77%)	104 (70.75%)	0.00*
3	63 (26.81%)	20 (22.73%)	43 (29.25%)	
**Lymphovascular invasion (**^**a**^**)**				
Yes	13 (6.05%)	5 (6.33%)	8 (5.8%)	0.02
No	202 (93.95%)	74 (93.67%)	128 (94.12%)	
**Ki-67 (**^**a**^**)**				
(+)	32 (14.16%)	16 (16.49%)	16 (13.97%)	0.13
(−)	201 (82.27%)	81 (83.51%)	120 (88.23%)	
Ki-67 (%) (^a^)	25.45 ± 6.10	27.44 ± 12.23	24.90 ± 13.49	0.42
**ER**				
(+)	232 (84.98%)	91 (83.49%)	141 (85.98%)	0.48
(−)	41 (15.02%)	18 (16.51%)	23 (14.02%)	
**PR**				
(+)	214 (79.55%)	85 (79.44%)	129 (79.63%)	0.29
(−)	55 (20.45%)	22 (20.56%)	33 (20.37%)	
**Cerb2 (**^**a**^**)**				
(+)	69 (28.28%)	22 (22.68%)	47 (31.97%)	0.23
(−)	175 (71.72%)	75 (77.32%)	100 (68.03%)	
NPI	3.64 ± 1.83	3.21 ± 1.81	3.71 ± 1.84	0.04
**NPI**				
< 2	43 (17.2%)	18 (19.35%)	25 (15.92%)	0.00*
2–2.4	44 (17.6%)	19 (20.43%)	25 (15.92%)	
2.4–3.4	48 (19.2%)	22 (23.66%)	26 (16.56%)	
3.4–5.4	47 (18.8%)	14 (15.05%)	33 (21.02%)	
> 5.4	68 (27.2%)	20 (21.51%)	48 (30.57%)	

Data expressed as means ± standard deviation and absolute numbers and their frequencies.

*NPI* Nottingham prognostic index.

*p value < 0.05 (t-Student or Chi-squared).

^a^Analysis performed on the cases provided by the pathology department.

**Table 7:** Prognostic tumor characteristics in patients according to their hormonal status (pre vs. postmenopausal).

Variable	All patients n = 347 (100%)	Premenopausal n = 108 (%)	Postmenopausal n = 239 (%)	*p*
Clinical tumor size (mm)	22.63 ± 14.64	23.31 ± 13.87	22.32 ± 15.02	0.61
Pathological tumor size (mm)	24.23 ± 14.87	24.04 ± 12.43	24.20 ± 15.63	0.93
**Tumor size (cm) (n = 68) (**^**a**^**)**				
≤ 2 cm	151 (57.85%)	49 (59.04%)	102 (57.30%)	0.86
> 2 cm	110 (42.15%)	34 (40.96%)	76 (42.70%)	
**Clinical stage**				
I	115 (41.22%)	36 (39.56%)	79 (42.02%)	0.65
II, III	143 (51.25%)	48 (52.75%)	95 (50.53%)	
III	21 (7.53%)	7 (7.69%)	14 (7.45%)	
**Pathological stage**				
Initial (I)	111 (36.75%)	33 (35.48%)	78 (37.32%)	0.95
Intermediate (II, III)	168 (55.93%)	52 (55.91%)	116 (55.50%)	
Advanced (IV)	23 (7.62%)	8 (8.61%)	15 (7.18%)	
**Axillary invasion (n = 48)**				
Negative	112 (39.44%)	40 (44.94%)	82 (40%)	0.53
Positive	172 (60.56%)	49 (55.06%)	123 (60%)	
**Histological type**				
Ductal	299 (95.22%)	101 (97.12%)	198 (94.29%)	0.18
Lobulillar	15 (4.78%)	3 (2.88%)	12 (5.71%)	
**Differentiation degree**				
1, 2	196 (71.01%)	58 (67.44%)	138 (72.63%)	0.80
3	80 (28.99%)	28 (32.56%)	52 (27.37%)	
**Lymphovascular invasion (**^**a**^**)**				
Yes	15 (6.38%)	5 (7.35%)	12 (7.10%)	0.26
No	220 (93.62%)	63 (92.65%)	157 (92.9%)	
**Ki-67 (**^**a**^**)**				
(+)	37 (13.55%)	9 (10.59%)	28 (14.89%)	0.60
(−)	236 (86.45%)	76 (89.41%)	160 (85.11%)	
Ki-67 (%) (^a^) n(84)	28.83 ± 12.11	28.74 ± 12.15	28.95 ± 13.14	0.94
**ER**				
(+)	269 (85.4%)	80 (83.33%)	189 (86.3%)	0.56
(−)	46 (14.6%)	16 (16.67%)	30 (13.7%)	
PR				
(+)	244 (77.96%)	78 (81.25%)	166 (76.5%)	0.25
(−)	69 (22.04%)	18 (18.75%)	51 (23.50%)	
**Cerb2 (**^**a**^**)**				
(+)	83 (29.12%)	28 (32.56%)	55 (27.64%)	0.63
(−)	202 (70.88%)	58 (67.44%)	144 (72.36%)	
NPI	3.71 ± 1.81	3.82 ± 1.76	3.59 ± 1.94	0.35
**NPI**				
< 2	49 (16.78%)	9 (10.11%)	40 (19.7%)	0.43
2–2.4	45 (15.51%)	14 (15.73%)	31 (15.27%)	
2.4–3.4	58 (19.85%)	22 (24.72%)	36 (17.73%)	
3.4–5.4	53 (18.15%)	17 (19.10%)	36 (17.73%)	
> 5.4	87 (29.79%)	27 (30.34%)	60 (29.56%)	

Data expressed as means ± standard deviation and absolute numbers and their frequencies.

*NPI* Nottingham prognostic index.

*p value < 0.05 (t-Student or Chi-squared).

^a^Analysis performed on the cases provided by the pathology department.

## Discussion

The main finding of this study was that compared to nonobese women, obese women with BC, especially BC patients with central adiposity, present several tumor factors indicating worse prognosis, regardless of menopausal status. On the other hand, we did not find an inverse relation between breast volume and tumor prognosis; rather, we observed a greater number of ER (+) tumors in patients with larger breasts.

Therefore, our findings are not in agreement with previous studies^22–26^, which conclude that women with larger breasts have more aggressive tumor characteristics than women with smaller breasts. In our study, we found a very consistent relationship between breast volume and BMI, but the relationship between breast volume and central obesity was less consistent. As stated before, central obesity (with hyperinsulinemia), not general obesity, could be associated with tumoral factors associated with worse prognosis. Thus, the volume of the breast would not be so related to the tumor prognosis because its relationship with the central adiposity is much lower. Most likely, the investigation of the fat/gland ratio of the breast or the mammographic density in these women could be of great value to assess the pathological risk in a more precise way^27^, but this was not part of this study.

The measurements in this study were taken before surgery by the same person, a nurse trained for this purpose, to minimize the risk of bias. We do not know if our results can be extrapolated to patients of other races or to another population with a higher prevalence of obesity. On the other hand, taking into account that many previous studies were carried out in groups of patients with a greater range of BMI than what was observed in our study population^3,10^, it is not clear whether the associations found here are linear or they would change in individuals with more extreme BMI values.

A plausible mechanism that could underlie the association between breast size and cancer prognosis may be an increase in IGF-1 levels^36^. Some studies have indicated a clear association between cancer and the insulin/IGF-1 axis^37–40^. Three of the studies demonstrated the participation of these factors in BC^38–40^. In conjunction with our finding of more ER (+) cancers in women with larger breasts, in the meta-analysis conducted by Key et al. in 2010, it was shown that the increase in IGF-1 levels was only associated with the risk of ER-positive BC^40^. In the follicular phase of the menstrual cycle, IGF-1 levels were positively associated with breast size in young null gravid women who did not use oral contraceptives^29^. In line with this finding, Hartmann et al^41^ showed that the success rate of breast augmentation resulting from estrogen stimulation was dependent on a subsequent increase in IGF-1 concentrations in women. On the other hand, high IGF-1 has been linked to mammographic density in premenopausal women, and mammographic density is significantly associated with mortality from BC^42,43^. According to these works, a larger breast size may therefore be a substitute marker for high levels of IGF-1. However, we cannot provide additional evidence since we have not analyzed the circulating levels of IGF-1 in our patients because it was not the objective of our study. Similarly, as we found that a large proportion of patients with larger breast sizes had ER-positive breast tumors, the measurement of estrogen levels in these patients would be an interesting point to be addressed, but we did not measure estrogen levels because this was not the objective of our study. A WHR > 0.85 was associated with more aggressive tumor characteristics in our study. A high WHR can be an indicator of a number of unfavorable conditions, such as a high testosterone/estrogen ratio^44^, increase in cortisol in response to stress or metabolic problems^17^ or hyperinsulinemia^18,45^.

Consequently, hyperinsulinemia (associated type II diabetes) associated with increased CHF could be important for the prognosis of BC. In mice, visceral fat has been shown to increase inflammation and aromatase expression in the mammary gland^46^. Measurements of circulating androgens, insulin, IGF-1, and cortisol may be beneficial for patients with a high WHR, as these measures may provide information regarding which pathway to target during BC treatment. There are ongoing trials with metformin^47^ and a phase II trial of nonsteroidal antiandrogen bicalutamide in women with ER (−)/PR(−)/AR(+) (androgen receptors) BC (ClinicalTrials.gov identifier NCT00468715).

Consistent with previous studies^5,48^, in our study, patients with a BMI ≥ 30 kg/m^2^ had larger tumors. Similar to Markkula et al^48^, we found no association between BMI ≥ 30 kg/m^2^ and hormone receptor status, in contrast to the results of Enger et al^10^. In the Enger study, only 73% of the tumors were ER positive, compared to more than 85% in the Markkula study and 85.4% in our study.

In one study^48^, despite finding larger tumors in the obese population, the researchers did not find a significant association between obesity and the prognosis of BC. This differs from the results of Petrelli et al^8^, who analyzed 2,852 deaths from BC in postmenopausal women with a follow-up of 14 years and found a worse vital prognosis among obese women.

## Conclusions

In conclusion, the present study demonstrates, in our environment, the relationship of obesity, especially central obesity, with several tumor biological factors indicating poor prognosis. On the other hand, in the global population (pre- and postmenopausal), we have not been able to find any association between breast volume and prognostic factors of BC, except for a greater proportion of ER (+) tumors in women with larger breast volumes. Menopausal status was not related to prognostic variables.

For future research, we believe that the acquisition of additional data is required to support our conclusions. In particular, the serum levels of insulin, IGF-1 and 17b-estradiol and their correlation with prognostic parameters in lean and obese patients should be assessed. This would support the role of central obesity in worse prognosis.

Our results justify the performance of a simple, fast and inexpensive anthropometric measurement (WHR) in mammary oncology clinical practice; this measure could provide important prognostic information beyond what is obtained through the report of pathology anatomy and clinical evaluation. Therefore, the results could be taken into account to adapt the intensity and modality of the treatment and follow-up of these patients with central obesity and to propose preventive treatments for the related and nonrelated morbidity and mortality (diabetes mellitus type II, metabolic syndrome, HTA). We believe we should continue investigating the possible relationship of breast volume with the prognosis of BC, especially in postmenopausal women with ER (+) BC.

## References

[1] Lahmann PH, Hoffmann K, Allen N (2004). Body size and breast cancer risk: findings from the European Prospective Investigation into Cancer and Nutrition (EPIC). Int. J. Cancer.

[2] Cui Y, Whiteman MK, Glaws JA (2002). Body mass index and stage of breast cancer at diagnosis. Int. J. Cancer.

[3] Han D, Nie J, Bonner MR (2006). Lifetime adult weight gain, central adiposity, and the risk of pre- and postmenopausal breast cancer in the Western New York exposures and breast cancer study. Int. J. Cancer.

[4] Carmichael AR (2006). Obesity and prognosis of breast cancer. Obes. Rev..

[5] Carmichael AR, Bates T (2004). Obesity and breast cancer: a review of the literature. Breast.

[6] Loi S, Milne RL, Friedlander ML (2005). Obesity and outcomes in premenopausal and postmenopausal breast cancer. Cancer Epidemiol. Biomark. Prev..

[7] Protani M, Coory M, Martin JH (2010). Effect of obesity on survival of women with breast cancer: systematic review and meta-analysis. Breast Cancer Res. Treat..

[8] Petrelli JM, Calle EE, Rodriguez C, Thun MJ (2002). Body mass index, height, and postmenopausal breast cancer mortality in a prospective cohort of US women. Cancer Causes Control.

[9] Pinheiro RL, Sarian LO, Pinto-Neto AM, Morais S, Costa-Paiva L (2009). Relationship between body mass index, waist circumference and waist to hip ratio and the steroid hormone receptor status in breast carcinoma of pre- and postmenopausal women. Breast.

[10] Enger SM, Ross RK, Paganini-Hill A, Carpenter CL, Bernstein L (2000). Body size, physical activity, and breast cancer hormone receptor status: results from two case-control studies. Cancer Epidemiol. Biomark. Prev..

[11] Suzuki R, Orsini N, Saji S, Key TJ, Wolk A (2009). Body weight and incidence of breast cancer defined by estrogen and progesterone receptor status—a meta-analysis. Int. J. Cancer.

[12] Healy LA, Ryan AM, Carroll P (2010). Metabolic syndrome, central obesity and insulin resistance are associated with adverse pathological features in postmenopausal breast cancer. Clin. Oncol. (R. Coll. Radiol.).

[13] Baumgartner KB, Hunt WC, Baumgartner RN (2004). Association of body composition and weight history with breast cancer prognostic markers: divergent pattern for Hispanic and non-Hispanic White women. Am. J. Epidemiol..

[14] Harvie M, Hooper L, Howell AH (2003). Central obesity and breast cancer risk: a systematic review. Obes. Rev..

[15] Borugian MJ, Sheps SB, Kim-Sing C (2003). Waist-to-hip ratio and breast cancer mortality. Am. J. Epidemiol..

[16] Jernstrom H, Barrett-Connor E (1999). Obesity, weight change, fasting insulin, proinsulin, C-peptide, and insulin-like growth factor-1 levels in women with and without breast cancer: the Rancho Bernardo Study. J. Womens Health Gend. Based Med..

[17] Bjorntorp P (1997). Hormonal control of regional fat distribution. Hum. Reprod..

[18] Hollmann M, Runnebaum B, Gerhard I (1997). Impact of waisthip-ratio and body-mass-index on hormonal and metabolic parameters in young, obese women. Int. J. Obes. Relat. Metab. Disord..

[19] Goodwin PJ, Ennis M, Pritchard KI (2002). Fasting insulin and outcome in early-stage breast cancer: results of a prospective cohort study. J. Clin. Oncol..

[20] Bruning PF, Bonfrer JM, van Noord PA, Hart AA, de Jong- BM, Nooijen WJ (1992). Insulin resistance and breastcancer risk. Int. J. Cancer.

[21] Del Giudice ME, Fantus IG, Ezzat S, McKeown-Eyssen G, Page D, Goodwin PJ (1998). Insulin and related factors in premenopausal breast cancer risk. Breast Cancer Res. Treat..

[22] Sellahewa C, Nightingale P, Carmichael AR (2008). Women with large breasts are at an increased risk of advanced breast cancer. Int. Semin. Surg. Oncol..

[23] Hsieh CC, Trichopoulos D (1991). Breast size, handedness and breast cancer risk. Eur. J. Cancer.

[24] Hall HI, Coates RJ, Uhler RJ (1999). Stage of breast cáncer in relation to body mass index and bra cup size. Int. J. Cancer.

[25] Ingram DM, Huang HY, Catchpole BN, Roberts A (1989). Do big breasts disadvantage women with breast cancer?. Aust. N. Z. J. Surg..

[26] Hoe AL, Mullee MA, Royle GT, Guyer PB, Taylor I (1993). Breast size and prognosis in early breast cancer. Ann. R. Coll. Surg. Engl..

[27] Sung J, Song YM, Stone J, Lee K, Kim SY (2010). Association of body size measurements and mammographic density in Korean women: the Healthy Twin study. Cancer Epidemiol. Biomark. Prev..

[28] Wade TD, Zhu G, Martin NG (2010). Body mass index and breast size in women: same or different genes?. Twin Res. Hum. Genet..

[29] Jernstro¨m H, Olsson H, (1997). Breast size in relation to endogenous hormone levels, body constitution, and oral contraceptive use in healthy nulligravid women aged 19–25 years. Am. J. Epidemiol..

[30] Ray JG, Mohllajee AP, van Dam RM, Michels KB (2008). Breast size and risk of type 2 diabetes mellitus. CMAJ.

[31] Schrauder MG, Fasching PA, Haberle L (2011). Diabetes and prognosis in a breast cancer cohort. J. Cancer Res. Clin. Oncol..

[32] Kusano AS, Trichopoulos D, Terry KL, Chen WY, Willett WC, Michels KB (2006). A prospective study of breast size and premenopausal breast cancer incidence. Int. J. Cancer.

[33] Ringberg, A., Bågeman, E., Rose, C., Ingvar, C. & Jernström, H. Of cup and bra size: reply to a prospective study of breast size and premenopausal breast cancer incidence. *Int. J. Cancer* **119**, 2242–2243 (2006); Author reply 4. 10.1002/ijc.22104 16841335

[34] Strombeck JO, Malm M (1986). Priority grouping in a waiting list of patients for reduction mammaplasty. Ann. Plast. Surg..

[35] Medical Association World (1997). Declaration of Helsinki: recommendations guiding physicians in biomedical research involving human subjects. JAMA.

[36] Jernstrom H, Sandberg T, Bageman E, Borg A, °, Olsson H, (2005). Insulin-like growth factor-1 (IGF1) genotype predicts breast volume after pregnancy and hormonal contraception and is associated with circulating IGF-1 levels: implications for risk of early-onset breast cancer in young women from hereditary breast cancer families. Br. J. Cancer.

[37] Pollak M (2008). Insulin and insulin-like growth factor signalling in neoplasia. Nat. Rev. Cancer.

[38] Hankinson SE, Willett WC, Colditz GA (1998). Circulating concentrations of insulin-like growth factor-I and risk of breast cancer. Lancet.

[39] Gee JM, Robertson JF, Gutteridge E (2005). Epidermal growth factor receptor/HER2/insulin-like growth factor receptor signalling and oestrogen receptor activity in clinical breast cancer. Endocr. Relat. Cancer.

[40] Key TJ, Appleby PN, Reeves GK, Roddam AW (2010). Insulinlike growth factor 1 (IGF1), IGF binding protein 3 (IGFBP3), and breast cancer risk: pooled individual data analysis of 17 prospective studies. Lancet Oncol..

[41] Hartmann BW, Laml T, Kirchengast S, Albrecht AE, Huber JC (1998). Hormonal breast augmentation: prognostic relevance of insulin-like growth factor-I. Gynecol. Endocrinol..

[42] Diorio C, Pollak M, Byrne C (2005). Insulin-like growth factor-I, IGF-binding protein-3, and mammographic breast density. Cancer Epidemiol. Biomark. Prev..

[43] Chiu SY, Duffy S, Yen AM, Tabar L, Smith RA, Chen HH (2010). Effect of baseline breast density on breast cancer incidence, stage, mortality, and screening parameters: 25-year follow- up of a Swedish mammographic screening. Cancer Epidemiol. Biomark. Prev..

[44] van Anders SM, Hampson E (2005). Waist-to-hip ratio is positively associated with bioavailable testosterone but negatively associated with sexual desire in healthy premenopausal women. Psychosom. Med..

[45] Peiris AN, Struve MF, Kissebah AH (1987). Relationship of body fat distribution to the metabolic clearance of insulin in premenopausal women. Int. J. Obes..

[46] Subbaramaiah K, Howe LR, Bhardwaj P (2011). Obesity is associated with inflammation and elevated aromatase expression in the mouse mammary gland. Cancer Prev. Res..

[47] Goodwin PJ, Stambolic V, Lemieux J (2011). Evaluation of metformin in early breast cancer: a modification of the traditional paradigm for clinical testing of anti-cancer agents. Breast Cancer Res. Treat..

[48] Markkula A, Bromée A, Henningson M, Hietala M, Ringberg A, Ingvar C, Rose C, Jernström H (2012). Given breast cancer, does breast size matter? Data from a prospective breast cancer cohort. Cancer Causes Control..

